# DC - SIGNR by influencing the lncRNA HNRNPKP2 upregulates the expression of CXCR4 in gastric cancer liver metastasis

**DOI:** 10.1186/s12943-017-0639-2

**Published:** 2017-04-13

**Authors:** Yu Zhang, Qianshi Zhang, Mengyang Zhang, Menglang Yuan, Zhaohui Wang, Jingbo Zhang, Xu Zhou, Yinan Zhang, Fang Lin, Heya NA, Shuangyi Ren, Yunfei Zuo

**Affiliations:** 1 Department of Clinical Biochemistry, College of Laboratory Diagnostic Medicine, Dalian Medical University, lvshun South Road West 9, Dalian, 116044 China; 2 Department of General Surgery, The Second Affiliated Hospital of Dalian Medical University, Dalian, Shahekou District Zhongshan Road no. 467, 116023 Dalian, China; 3 Department of Clinical Laboratory, The Second Affiliated Hospital of Dalian Medical University, Dalian, 116023 China

**Keywords:** Gastric cancer, Liver metastasis, DC-SIGNR, lncRNA HNRNPKP2, STAT5A, CXCR4

## Abstract

**Background:**

Profiling evidences of selectin demonstrate that they play an crucial role in cancer progression and metastasis. However, DC-SIGNR as a family member of selectin participates in gastric cancer liver metastasis remains unknown.

**Methods:**

The serum level of DC-SIGNR was evaluated in gastric cancer patients by ELISA. Manipulation DC-SIGNR expression in BGC823 and SGC7901 cell lines was mediated by lentivirus. Investigation the biological effects of DC-SIGNR were verified by MTT, wounding and transwell in vitro and experiments on animals to confirm gastric cancer liver metastasis by IVIS. Insights of the mechanism were employed microarray and bioinformatic analysis. Further to confirm the results were conducted by qRT-PCR, western blot and by flow cytometry.

**Results:**

DC-SIGNR serum level was significantly increased in gastric cancer patients compared with healthy group. Additionally, DC-SIGNR level was associated with an advanced pathological stage in gastric cancer patients. DC-SIGNR knockdown inhibited the proliferation, migration and invasion of gastric cancer cells in vitro and suppressed the liver metastasis in vivo. While, DC-SIGNR overexpression promoted cell proliferation, migration and invasion. In mechanism, HNRNPKP2 as a lncRNA was upregulated after DC-SIGNR knockdown. Importantly, STAT5A promoted HNRNPKP2 expression after knockdown DC-SIGNR. Furthermore after HNRNPKP2 depletion, the downstream target gene CXCR4 was downregulated.

**Conclusions:**

DC-SIGNR promoted gastric cancer liver metastasis mediated with HNRNPKP2 which expression was regulated by STAT5A. And HNRNPKP2 decreased the expression of downstream target gene CXCR4. These findings indicated potential therapeutic candidates for gastric cancer liver metastasis.

**Electronic supplementary material:**

The online version of this article (doi:10.1186/s12943-017-0639-2) contains supplementary material, which is available to authorized users.

## Background

Gastric cancer (GC) is the second leading cause of cancer-associated mortality worldwide and incidence rates are highest in Eastern Asia, Latin America, Central and Eastern Europe [1, 2]. In China, gastric cancer is also a main malignant tumour and a chief reason of cancer deaths. The majority of GC patients are diagnosed at an advanced stage, 5-year survival rate of 11–42%. The prime determinant of survival following gastric carcinoma appears to be the development of liver metastasis [3, 4]. Despite surgical resection and chemoradiotherapy can control most cancer cells [5], a surgical resection has been rarely indicated for liver metastasis from gastric cancer [6]. So far, the complex molecular mechanism of liver metastasis has still remained essentially unknown. Therefore, we need to explore novel molecules to better understand the mechanism of hematogenous metastasis.

Metastatic spreading and the formation of secondary neoplasms from primary site are not random, exhibiting organ selectivity [7]. Recently the roles of intrinsic cancer cell properties have been investigated, such as selectin. In experimental metastasis studies, researchers demonstrate that liver sinusoidal endothelial cell lectin (LSECtin) mediated colon cancer cells metastasis to liver displays enhanced abilities to the specific organ [8]. Also, serum of soluble E-selectin (sE-selectin) concentration in gastric cancer patients are detected by ELISA, but increasing only in gastric cancer patients with peritoneal metastasis [9]. Similarly, hepatic sinusoidal endothelial E-selectin expression is up regulated by highly metastatic cells entering the liver [10]. Moreover, using an E-selectin-specific monoclonal antibody reduces liver metastasis, showing that E-selectin is involved in metastatic formation in this organ [11]. For further study, blocking colorectal carcinoma-induced hepatic endothelial E-selectin expression inhibits liver metastasis [12]. These events suggest that selectin play a key role in tumour metastasis to the target organs. DC-SIGNR (DC-SIGN-related protein, also known as L-SIGN, CD299) as a member of C-type lectin belonging to selectin is found high serum concentration in colon cancer patients [13]. Here, we ask whether DC-SIGNR contributes to hematogenous metastasis from gastric carcinoma.

Long ncRNAs (lncRNAs) which lengths are more than 200 nt are abundant in the human genome [14]. Recently, several long ncRNAs have been reported to have a role in gastric cancer metastasis. The lncRNA HULC is higher expression in GC tissues than pair-matched adjacent normal tissues and is significantly associated with distal metastasis and lymphatic metastasis [15]. While, FENDRR, as a tumour suppressor lncRNA, is downregulated in GC tissues and cell lines. Overexpression FENDRR exhibits the inhibiting capacity for cell migration and invasion in vitro and effectively reduces the number of metastatic nodules in vivo [16]. According to situ hybridization analysis and microarray data, the lncRNA GAPLINC is associated with GC proliferation, migration and angiogenesis. These functions are reduced by CD44 repression [17]. Another two extensively studied lncRNAs are HOTAIR and H19. They are correlated with GC development and poor prognosis. Gain and loss function analysis have showed that HOTAIR and H19 can drive gastric cell lines proliferation, migration and invasion [18, 19].

Signal transducer and activator of transcription (STAT) molecules are ubiquitously expressed in many tumour, such as breast cancer, lung cancer and head and neck cancer [20]. Interestingly, STAT family members can migrate to the nucleus to regulate gene expression at transcription level. Zhu et al. addressed that STAT5 controls some genes expression by binding to promoter sequences [21]. CXCR4 is ubiquitously expressed in many human cancer cell lines [22]. It binds to unique ligand CXCL12. So CXCR4 positive tumour cells move towards the high concentration CXCL12 organs to realize tumour metastasis.

In this study, DC-SIGNR was significantly increased in serum of gastric cancer patients and increased in middle-late patients. Then we characterized that DC-SIGNR promoted the biological function proliferation, migration and invasion on GC cell lines in vitro and promoted GC cells liver metastasis in vivo. Next, we employed LncPath chip to investigate the mechanism of DC-SIGNR mediated gastric cancer liver metastasis by the lncRNA HNRNPKP2. HNRNPKP2 was upregulated after DC-SIGNR knockdown. Furthermore, STAT5A promoted HNRNPKP2 expression after knockdown DC-SIGNR and CXCR4 was obviously decreased after knockdown HNRNPKP2. Collectively, we firstly found that DC-SIGNR was increased in gastric cancer patients serum and DC-SIGNR facilitated gastric cancer liver metastasis. A novel lncRNA HNRNPKP2 regulated by STAT5A was influenced by DC-SIGNR, and then DC-SIGNR promoted the expression of CXCR4.

## Methods

### Serum collection

Serum of gastric cancer patients and healthy samples were obtained from the First Hospital affiliated to Dalian Medical University and the Second Hospital affiliated to Dalian Medical University between 2013 and 2014. All gastric cancer cases were reviewed by pathologists and histologically confirmed as gastric cancer (stageI,II, III, IV; 7th Edition AJCC) based on histopathological evaluation. Clinical pathology information was available for all samples (Additional file 1: Table S1). No local or systemic treatment obtained from all patients. Recombinant human DC-SIGNR Fc chimera was used as standard substance (R&D Systems). The protocols and procedures were approved by the Dalian Medical University research ethics committee. Written informed consent was obtained from all patients. The standard curve of sDC-SIGNR was listed in Additional file 2: Figure S1.

### Cell lines and culture conditions

Four gastric cancer cell lines (BGC823, SGC7901, MGC803 and HGC27), three colon cancer cell lines (SW620, LoVo and HCT116), three breast cancer cell lines (MCF-7, MDA-MB-231, MDA-MB-435), two liver cancer cell lines (PLC/PRF/5 and HepG2) and one human normal liver cell line (LO2) were purchased from the Institute of Biochemistry and Cell Biology of the Chinese Academy of Sciences (Beijing, China). Cells were cultured in RPMI 1640 or DMEM (Hyclone) medium supplemented with 10% fetal bovine serum (tBD Bioscience), 100 U/ml penicillin, and 100 mg/ml streptomycin (Beyotime) in humidified air at 37 °C with 5% CO_2_.

### RNA extraction and qRT-PCR analyses

Total RNA was extracted from formalin fixed and paraffin embedded gastric cancer tissues using RNAprep pure FFPE kit (TIANGEN). Total RNA was extracted from cultured cells using TRIZOL reagent (TaKaRa Bio, Otsu, Japan). For qRT-PCR, RNA was reverse transcribed to cDNA by using random primer (TIANGEN Biotech). QRT-PCR analyses were performed with Power SYBRGreen (TaKaRa). Results were normalized to the expression of GAPDH. The primers were listed in Additional file 3: Table S3. QRT-PCR and data collection were performed on Thermal Cycler Dice Real Time System (TaKaRa Bio, Otsu, Japan)

PCR reactions were performed at 94 °C for 2 min, followed by 41 cycles of 94 °C for 15 s and 58 °C for 20 s. ΔCt was calculated by subtracting the Ct of internal control RNA from the Ct of lncRNA or the mRNA of interest, respectively. ΔΔCt was then calculated by subtracting the ΔCt of the internal control from the ΔCt of the target group. Fold change of DC-SIGNR or HNRNPKP2 was calculated by the equation 2^-ΔΔCt^.

### Ectopic expression and knockdown of DC-SIGNR

Based on the expression of DC-SIGNR in GC cell lines, we selected SGC7901 cells for the enhanced expression study and BGC823 cells for the knockdown study. For ectopic expression, the full-length DC-SIGNR cDNA was subcloned into the LV5 lentiviruses (GenePharma, Suzhou) and infected into SGC7901 cells to generate *SGC7901/DC-SIGNR*.

For knockdown of DC-SIGNR expression, two complementary oligonucleotides of small hairpin RNA sequences were chemically synthesized, subcloned into the pGLV3 lentiviruses (GenePharma, Suzhou) and infected into BGC823 cells to generate *BGC823/DC-SIGNR shRNA*.

Briefly, a total of 3 × 10^4^ cells were plated in 24-well plates for 24 h and then infected with the lentiviral vectors described above by means of polybrene (GenePharma, Suzhou) for 48 h. Concentration of puromycin (SIGMA) on SGC7901 and BGC823 is 0.6 μg/ml to get stably transfected cell lines. The negative control (GenePharma, Suzhou) was infected in parallel. The cells were then subjected to RNA extraction and further functional assays.

Knockdown HNRNPKP2, knockdown STAT5A and ectopic expression of STAT5A. Knockdown HNRNPKP2 was developed by transfecting siRNAs (GenePharma, Suzhou) against HNRNPKP2 into BGC823/DC-SIGNR shRNA to generate BGC823/DC-SIGNR shRNA/siHNRNPKP2. Negative siRNAs (GenePharma, Suzhou) were transfected as negative control and termed BGC823/DC-SIGNR shRNA/NC.

Knockdown STAT5A in SGC7901/DC-SIGNR cells was produced by transfecting siSTAT5A (GenePharma, Suzhou) and named SGC7901/DC-SIGNR shRNA/siSTAT5A. Negative siRNAs (GenePharma, Suzhou) were transfected as negative control and termed SGC7901/DC-SIGNR shRNA/NC.

For ectopic expression of STAT5A, pCMV3-Flag-STAT5A (CWBIO, Beijing) and pCMV3-empty were transfected into BGC823/DC-SIGNR shRNA.

Briefly, a total of 3 × 10^6^ cells were plated in 6-well plates for 24 h and then transfected with the siRNA or plasmid described above by means of Lipofectamine 2000 (Invitrogen). The cells were then subjected to RNA extraction after 24 h and to protein extraction after 48 h for further functional assays. Target sequences for DC-SIGNR knockdown or overexpression, for HNRNPKP2 siRNA and for STAT5A siRNA were listed in Additional file 4: Table S4.

### Cell proliferation assay

Cell proliferation was monitored by ELISA with MTT kit (Biosharp) according to the manufacturer’s instruction. Briefly, 100ul of cell suspension from each subgroup (2,000 cells/well) was placed in a 96-well plates and pre-incubated for 12 h. Then, 20 μl of MTT solution was added to each well and incubated for 4 h until purple precipitate was visible. After adding detergent agent for 2 h, the number of cells was counted every 24 h for 5 days by measuring the absorbance at 490 nm using Microplate (BIO-RAD). For colony formation assays, cells were placed into 6-well plates and maintained in media containing 10% FBS for 2 weeks. Colonies were fixed with methanol and stained with 0.1% crystal violet. Visible colonies were manually counted.

### Flow-cytometric analysis of BGC823 and SGC7901

BGC823 cells and SGC7901 cells were harvested by trypsinization. After staining with CD299-APC (eBioscience) and CXCR4-PE (BioLegend), cells were analyzed with a flow cytometry (FACScan®; BD Biosciences) equipped with a Summit 5.0 software.

### Wound-healing assay

For the wound healing assay, a total of 6 × 10^5^ cells were seeded in 6-well plates and cultured overnight until 90% confluent. A sterile yellow pipette tip was used to make a straight scratch. The suspension cells were washed off twice gently and images of the scratch were acquired as baseline. The medium was then replaced and images of the same location were obtained using a microscope for next days.

### Cell migration assay and invasion assays

Cell migration was assessed using transwell chamber of 8-μm pore size (Corning) according to the manufacturer’s instruction. Briefly, 200 μl of serum-free medium containing 3 × 10^5^ cells from each subgroup were added to the upper chamber. For the invasion assays, 4 × 10^5^ cells in 200 μl serum-free medium were added to the upper chamber coated with Matrigel (BD Matrigel^TM^). A volume of 0.6 ml of 10% FBS-containing medium was then added to the lower chamber as a chemoattractant. Cells were incubated for another 24 h at 37 °C in 5% CO_2_. After the incubation, cells on the upper surface of the membrane were scraped off with cotton swabs. Cells migrated to the bottom of the membrane were fixed and stained with 0.1% Crystal Violet Staining Solution. The cells on the bottom of the membrane were counted from five different microscopic fields and the average number was calculated.

### Metastasis assay in athymic nude mice model

4 ~ 6-week-old athymic BALB/C nude mice were purchased from Yangzhou University. For xenograft models, BGC823 cells infected negative control were collected at growing stage when confluence was about ~80%. 2 × 10^6^, 4 × 10^6^, 6 × 10^6^, 8 × 10^6^, 1 × 10^7^ BGC823 cells were injected subcutaneously into the right flanks of the five BALB/C nude mice. The state of the mice were observed every three days, including tumour volumes. Fifteen days after implantation, mice were sacrificed and tumour were removed out. The formula V (mm^3^) = A × B^2^/2 was applied to calculate the volume (A means the largest diameter, B means the perpendicular diameter).

For metastasis assays, 5 × 10^6^ BGC823 cells with DC-SIGNR knockdown (n =4 per group) and negative control (n =4 per group) in 100 μl PBS were inoculated into spleen of mice. At the first sigh of suffering post-inoculation, tumour in situ and hepatic metastasis were removed for IVIS (Carestream Health, Inc.) and sliced for IHC. The protocol was approved by the Committee on Ethics of Animal Experiments of the Dalian medical University.

### Immunohistochemistory (IHC)

Paraffin-embedded, formalin-fixed tissues of mice liver and spleen were immunostained for Ki67, CK7 and CK20 proteins. The signal was amplified and visualized with diaminobenzidine-chromogen, followed by counterstaining with hematoxylin. Anti-Ki67 (1:50), anti-CK7 (1:50) and anti-CK20 (1:50) were purchased from Proteintech (CK7 and CK20) and Bioworld (Ki-67).

### Microarray analysis

Samples preparation and microarray hybridization were performed by Kangchen Bio-tech, Shanghai P.R. China. Briefly, RNA was extracted from cultured flask at a concentration of 1 × 10^7^ cells using TRIZOL reagent (TaKaRa Bio, Otsu, Japan). Then, each sample was amplified and transcribed into fluorescent cRNA along the entire length of the transcripts without 3’ bias utilizing a random priming method. The labeled cRNAs were hybridized onto the LncRNA^TM^ Arrays (6 × 7 K, Arraystar). After having washed the slides, the arrays were scanned by the Axon GenePix 4000BScanner. GenePix Pro software (version 6.0) was utilized to analyze acquired array images. Quantile normalization and subsequent data processing were performed using the R limma software package.

### Western blot assay

The protein was extracted from cultured cells using a total protein extraction kit (KeyGen, Nanjing, China) and the protein concentration of the lysates was measured using the BCA Protein Assay Kit (Beyotime Biotechnology, Shanghai, China).

Equivalent amounts of protein was electrophoresed in 8% SDS polyacrylamide gels for STAT5A protein and 12% SDS polyacrylamide gels for β-actin protein, transferred onto 0.22 μm nitrocellulose membranes (Pall corporation). The membranes were incubated with specific antibodies and corresponding secondary antibodies. Signals were detected with electrochemical luminescence reagent (Advansta, USA). β-actin was used as control, anti-STAT5A (1:500) was purchased from Elabscience.

### Bioinformatic analysis

The online software WebGestalt (http://www.webgestalt.org/) was used to assessment target genes biological insights.

### Statistical analysis

Student’s *t*-test (two-tailed) and one-way ANOVA test were performed to analyze the in vitro and in vivo data using GraphPad Software. P values less than 0.05 were considered significantly.

## Results

### DC-SIGNR is increased in gastric cancer serum and correlates with advanced pathological stage

**Fig. 1:**
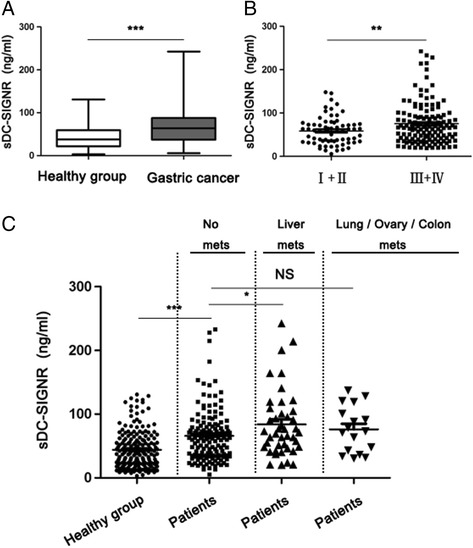
The level of DC-SIGNR in gastric cancer patients and its clinical significance. **a**, the level of DC-SIGNR in gastric cancer patients serum (*n* = 207) compared with healthy group (*n* = 197). DC-SIGNR level was examined by ELISA. **b**, examination of the correlation between DC-SIGNR level and advanced pathological stage. **c**, DC-SIGNR in healthy group (*n* = 197); patients with no metastasis (*n* = 151), liver metastasis (*n* = 43), or lung, ovary and colon metastasis (*n* = 17)

ELISA analysis was used to examine the DC-SIGNR level in 207 gastric cancer serum and 197 healthy group. DC-SIGNR expression was significantly increased (P < 0.0001) in gastric cancer serum compared with healthy group (Fig. 1a). Then we evaluated the correlation of DC-SIGNR expression with clinicopathological parameters to assess its clinical significance. As presented in Fig. 1b, middle-late patients which presented a higher tumour burden and more advanced tumour were associated with increased DC-SIGNR level. However, no correlation with age, gender and gastric cancer marker CA199 was observed (Table 1). DC-SIGNR level was correlated with advanced TNM staging (Fig. 1b). DC-SIGNR was significantly increased in gastric cancer patients with liver metastasis compared with patients with no metastasis (Fig. 1c). Taken together, our data indicated that DC-SIGNR correlated with liver metastasis in gastric cancer.

**Table 1:** Correlation of the expression of DC-SIGNR with clinicopathological features

Clinicopathological features	Number		DC-SINGR expression^a^	*P*-value^b^
Gender				
Male	159		68.57 ± 3.490	0.422
Female	48		74.31 ± 5.895	
Age				
<65	126		67.41 ± 3.594	0.302
≥65	81		73.78 ± 5.273	
Stage				
T1	15		51.59 ± 6.554	0.038
T2	23		52.95 ± 5.010	
T3	36		76.65 ± 8.575	
T4	102		69.95 ± 4.122	
Tx	31		83.33 ± 9.121	
Invasive-lyph node				
N0	47		60.18 ± 4.934	0.024
N1	40		62.25 ± 5.088	
N2	43		65.34 ± 5.896	
N3	41		78.84 ± 7.813	
Nx	36		86.37 ± 9.302	
Invasive-metastasis				
M0	151		65.82 ± 3.283	0.025
M1	56		80.92 ± 6.551	
CEA				
≤5	130	no check 23	64.18 ± 3.259	0.0008
>5	54		87.34 ± 7.092	
CA199				
≤35	140	no check 29	69.76 ± 3.668	0.225
>35	36		79.55 ± 6.977	

^a^Mean ± SEM

^b^ *P* < 0.05 was considered significant (Student`s *t* test between 2 groups and one-way ANOVA test for 3 groups)

### Manipulation of DC-SIGNR level in BGC823 and SGC7901

**Fig. 2:**
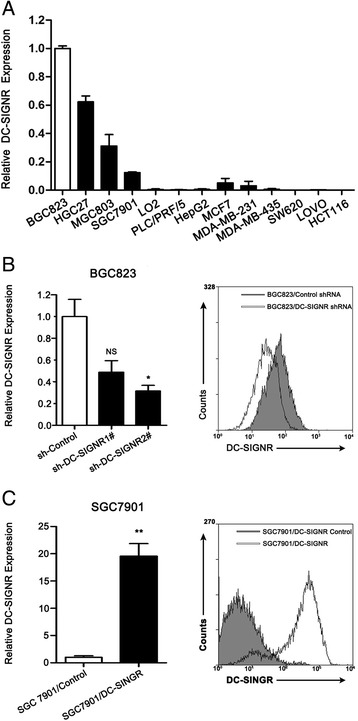
The expression of DC-SIGNR in various cell lines and manipulate the level of DC-SIGNR expression in gastric cancer cell lines. **a**, qRT-PCR was performed to demonstrate DC-SIGNR expression in various cell lines. **b** qRT-PCR (left panel) and flow cytometry assay (right panel) were used to confirm the knockdown efficiency of BGC823 infected with sh-DC-SIGNR. **c** qRT-PCR (left panel) and flow cytometry assay (right panel) were used to confirm the overexpression efficiency of SGC7901 infected with DC-SIGNR mediated by lentivirus

DC-SIGNR was expressed in the endothelial cells of the lymph nodes and liver. First, we determined DC-SIGNR expression level in different cell lines and in parallel the expression of DC-SIGNR by qRT-PCR, normalized to GAPDH. As shown in (Fig. 2a), DC-SIGNR gene was ubiquitously expressed in gastric cancer cell lines, breast cancer cell lines, liver cancer cell lines and normal hepatic epithelium cell line (LO2). There was no expression of DC-SIGNR in colon cancer cell lines. Furthermore, to investigate potential functional role of DC-SIGNR in gastric cancer cell lines, we manipulated DC-SIGNR level in BGC823 and SGC7901 cell lines. We performed knockdown DC-SIGNR in BGC823 by lentivirus infection with DC-SIGNR shRNA, while overexpression DC-SIGNR in SGC7901 by lentivirus infection with DC-SIGNR sequence. QRT-PCR assays revealed that DC-SIGNR expression was significantly reduced after infection with sh-DC-SIGNR, meanwhile overexpression DC-SIGNR was sharply increased after infection with DC-SIGNR sequence (Fig. 2b and c). Next, flow cytometric analysis was performed to further examine whether we successfully manipulated DC-SIGNR level. As shown in Fig. 2b and c, we got the consistent results. Therefore, we chose BGC823 and SGC7901 with different expression DC-SIGNR for next study.

### DC-SIGNR promotes gastric cancer cells proliferation,migration and invasion in vitro

**Fig. 3:**
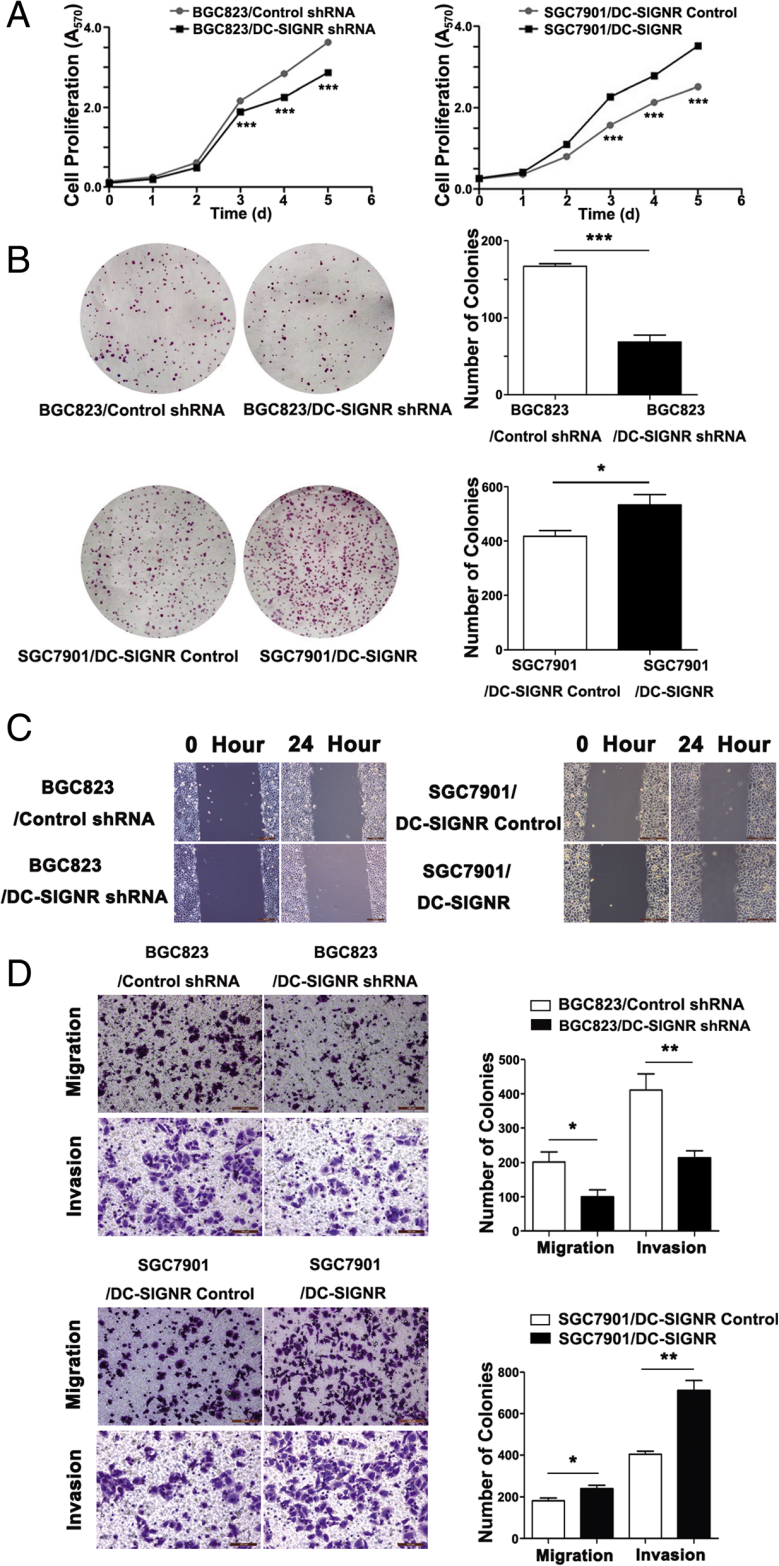
The function of DC-SIGNR in vitro. **a**, cell proliferation was measured by MTT assay. BGC823 cells were infected with sh-DC-SIGNR. SGC7901 cells were infected with DC-SIGNR mediated by lentivirus. **b** colony-forming growth assays were performed to determine the proliferation of sh-DC-SIGNR infected BGC823 cells and DC-SIGNR mediated by lentivirus infected SGC7901 cells. Colonies were counted and captured. **c** wound-healing assays were conducted to determine the rate of migration by measuring the distance from one edge of the wound to the other side. **d** transwell assays were conducted to investigate changes in BGC823 cells and SGC7901 cells migration and invasion. All experiments were performed in biologic triplicates with three technical replicates; *P < 0.05, **P < 0.01 and ***P < 0.001

To assess the possible biological importance of DC-SIGNR in tumorigenesis of gastric cancer, we observed its effect on cell proliferation. As shown in Fig. 3a, MTT assay revealed that DC-SIGNR knockdown inhibited BGC823 cells proliferation compared to the negative control, while DC-SIGNR overexpression got the opposite effect in SGC7901 cells. Furthermore, cell proliferation was also measured using a colony formation assay. Compared with the control cells, DC-SIGNR knockdown in BGC823 cells remarkably decreased colony formation abilities (P = 0.0005), whereas DC-SIGNR overexpression in SGC7901 cells increased clonogenic survival (p = 0.039) (Fig. 3b). These findings indicated that DC-SIGNR may be closely associated with the proliferation of gastric cancer cell lines. Invasion as metastasis cascade was acquired in malignant progression. We further assessed the effects of DC-SIGNR on cell migration and invasion. Transwell assay revealed that migration and invasion potential were significantly decreased after inhibition of DC-SIGNR in BGC823 cells. By contrast, overexpression DC-SIGNR exhibited strong ability to migration and invasion in SGC7901 cells (Fig. 3d). In the wound-healing assay, the scratch healed slower in BGC823 cells with knockdown DC-SIGNR than in the negative control. Conversely, healing was faster in SGC7901 cells with overexpression DC-SIGNR (Fig. 3c). Taken together, transwell analysis and the wound-healing analysis results suggested that DC-SIGNR exerted a critical effect on gastric cancer cell migration and invasion.

### DC-SIGNR promotes gastric cancer liver metastasis in vivo

**Fig. 4:**
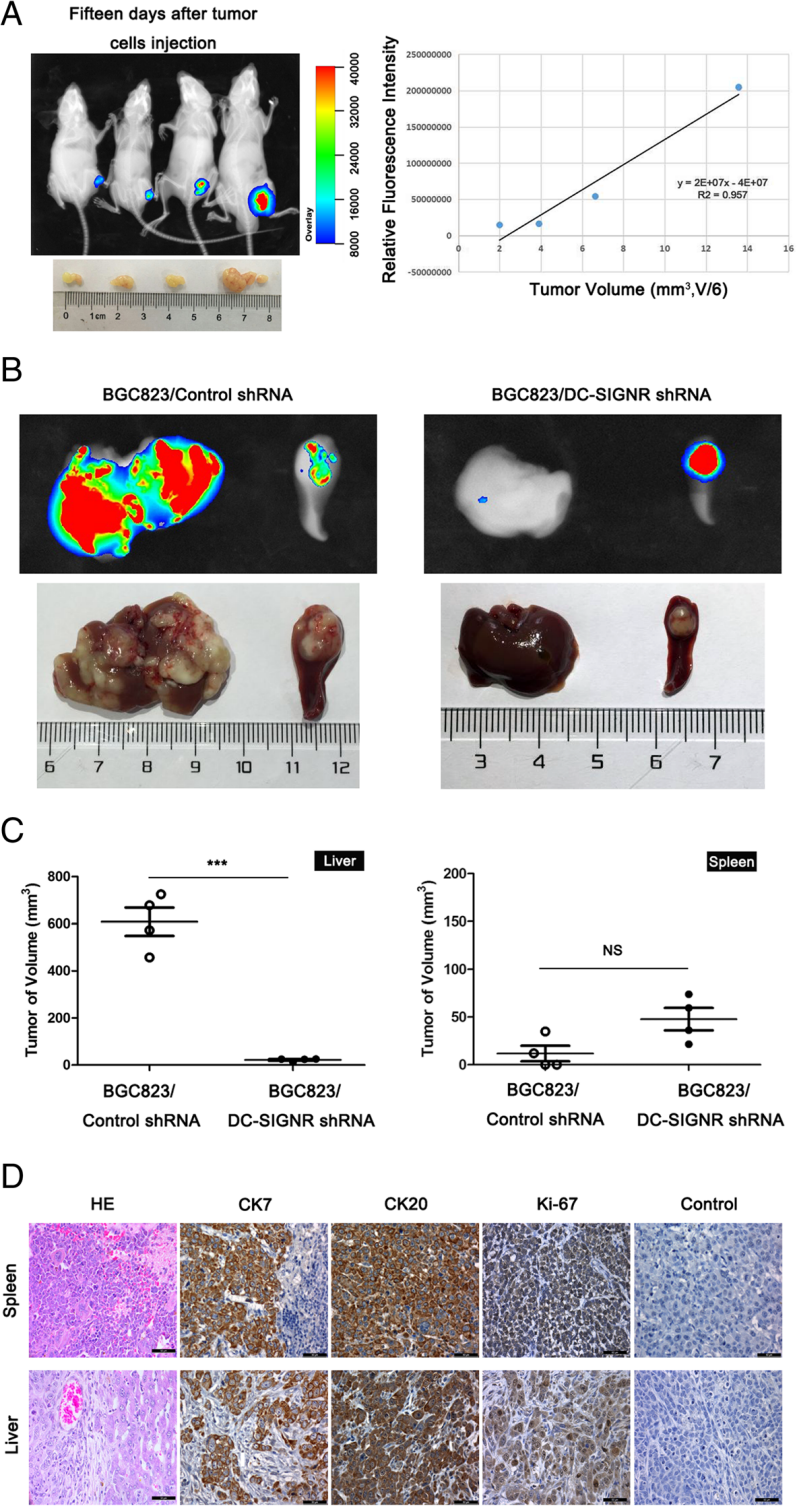
The effects of DC-SIGNR knockdown on gastric cancer liver metastasis cascade in vivo. **a** images of GFP signals of mice 15 days after subcutaneous injection with different concentrations of BGC823 cells infected negative control (left panel). The relationship between tumour volume and fluorescence intensity (right panel). **b** representative images of GFP signals of mice in each group after intrasplenic injection with 5 × 10^6^ BGC823 cells after knockdown DC-SIGNR and photographs of tumours after dissection. **c** average volume of tumours on liver and spleen derived from BGC823 cells after infected DC-SIGNR and negative control (left panel) and average volume of tumours on spleen derived from BGC823 cells after infected DC-SIGNR and negative control (right panel). **d** H&E, CK7, CK20 and Ki-67 staining of sections from liver tumours and spleen tumours were performed (scale bar = 50 μm)

Green fluorescent protein applied for tumour-bearing nude mouse for in vivo. Then we observed whether the fluorescence intensity connected with the tumour volume. Different concentration green fluorescently labelled BGC823 were injected into subcutaneous of nude mice and monitored tumour growth everyday. After fifteen days, the mice were taken X-Ray photo and fluorescent photo. The results were indicated that tumour size was closely relative to the total fluorescence intensity (Fig. 4a).

To examine whether gastric carcinoma colonize specific organ site liver also correlated with the presence of DC-SIGNR, green fluorescently labelled BGC823 cells with DC-SIGNR knockdown compared to negative control were injected into the spleens of nude mice. At the beginning to show symptoms of dying after the injection, quantfied tumour metastasis to distant organs by in-vivo Imaging System (Fig. 4b). We observed that inhibition of DC-SIGNR decreased gastric carcinoma liver metastasis. After calculated the mean volumes of the liver metastasis, there was a significantly decrease in carcinoma volume in the DC-SIGNR knockdown group compared with the negative group (Fig. 4c). Then H&E staining and immunohistological staining of Ki-67, CK7 and CK20 were used to verify gastric tumour invaded normal spleen and liver of the mice compared with negative control mice (Fig. 4d). Taken together, our data suggested that DC-SIGNR promoted tumorigenicity and metastasis.

### LncRNA expression profiling

**Fig. 5:**
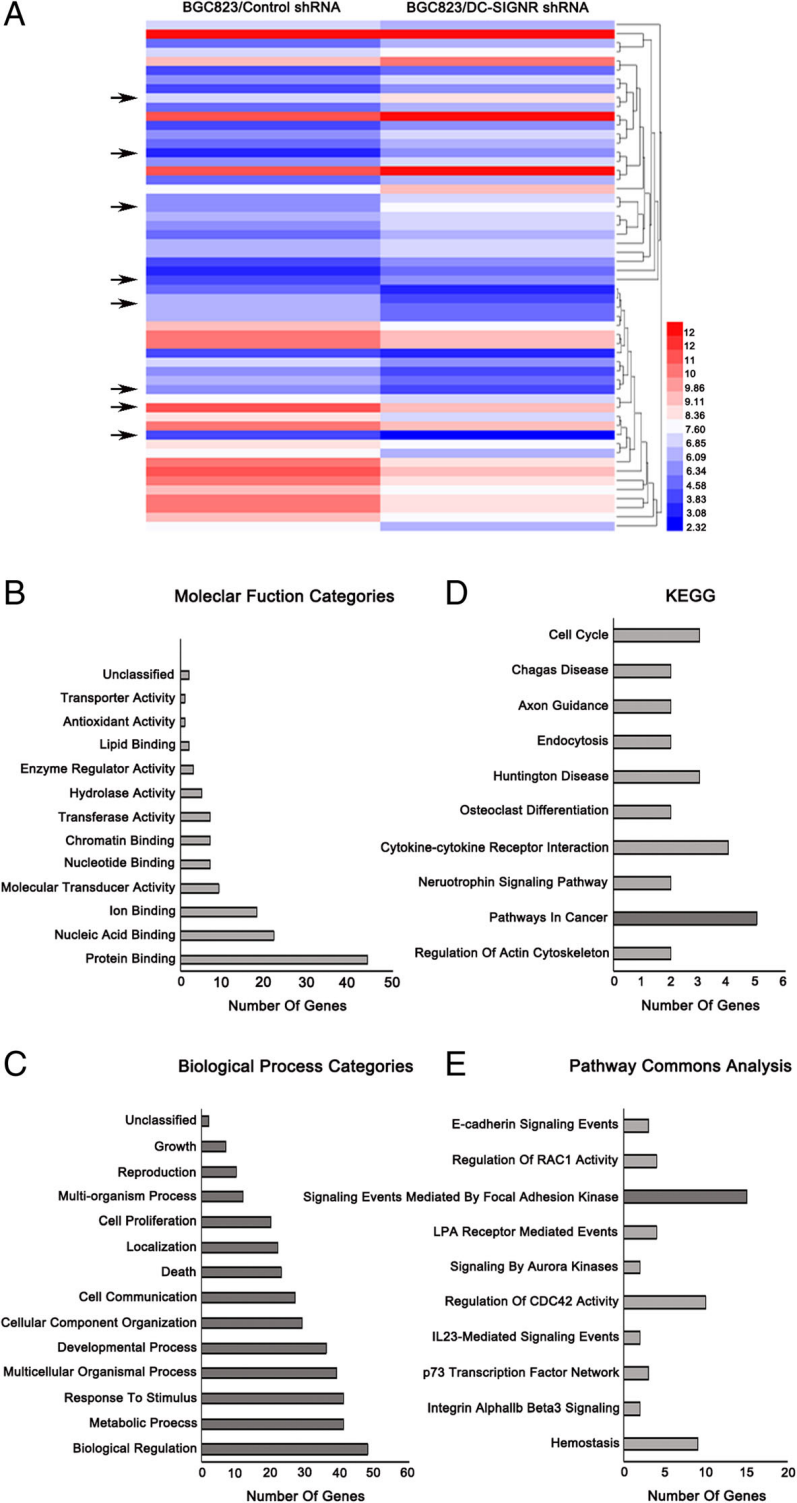
DC-SIGNR knockdown leads to cancer-related lncRNA expression profiling. **a**, the hierarchical cluster heatmap demonstrated lncRNAs expression with change fold > 2 from microarray data. **b**-**c**, bar graphs analyzed differentially expressed genes based on GO Slim with molecular function and biological processes. **d**-**e** KEGG and Pathway commons analysis revealed enrichment of genes related to various events

**Fig. 6:**
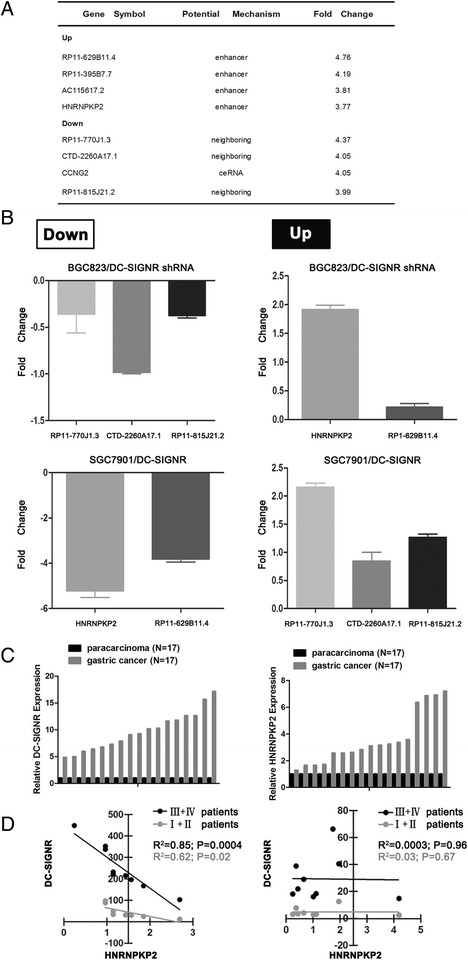
Five gene expression profiling with DC-SIGNR level change and correlation between DC-SIGNR and HNRNPKP2. **a** top 8 genes significantly upregulated or downregulated in BGC823 cells after DC-SIGNR knockdown. **b** real-time PCR confirmed the 8 genes expression using the gene specific primers. **c** relative expression of DC-SIGNR and HNRNPKP2 in gastric FFPET (*n* = 17) and paracarcinoma FFPET (*n* = 17) was detected by real-time PCR. **d** Correlation DC-SIGNR expression and HNRNPKP2 expression inI,IIgastric FFPET and III,IVgastric FFPET (left). And in I,II paracarcinoma FFPET and III,IVparacarcinoma FFPET (right). FFPET, formalin-fixed paraffin-embeded tissue

There is increasing interest in the role of lncRNAs in gastric cancer pathogenesis, including metastasis. And some researchers have confirmed that lncRNAs from microarray profiling play crucial roles in GC and aggressive progression [15–19]. Functional miroRNAs as regulators participate in DC-SIGN expression, forming microRNA-protein network, such as overexpression miR-511, miR-99b and miR-155 decreased DC-SIGN at the mRNA and protein level [23, 24]. Some lncRNAs interact with microRNAs to prevent the mRNA degradation [25]. Or some lncRNAs act as microRNAs sponges to interact with microRNAs to reduce microRNA influence on their target mRNAs [26]. DC-SIGNR and DC-SIGN were both belonging to C-type lectin family, so we employed LncPath chip analysis to identify lncRNA that exhibited a change in expression after DC-SIGNR knockdown in BGC823 cells. With 2829 probes, the profiling analysis identified 56 lncRNAs as being significantly differentially expressed (at least a two-fold change in expression in cells after DC-SIGNR knockdown compared with control group). Of the differentially expressed lncRNA, there were 28 lncRNAs upregulated by at least 2 fold changes, and 28 lncRNAs downregulated by 2 fold changes. The top eight different lncRNAs were listed in Fig. 5a and 6a, summarized by the average fold change (a complete list was shown in Additional file 5: Table S2).

For lncRNA, there were 57 target genes as downstream moleculars and 57 genes can unambiguously map to 53 unique Entrez Gene IDs (a complete list was shown in Additional file 6: Table S5). We applied WebGESTALT for biological insights [27]. Molecular function of Gene Ontology (GO) slim classification showed that protein binding function category had the most different expressed genes (Fig. 5b). With DC-SIGNR knockdown, biological processes had been changed, including cell proliferation and cell grow (Fig. 5c). For enrichment of genes associated with disease, 8 different genes were dysregulated in neoplastic processes (Fig. 5d). Meanwhile, pathway analysis provided one of the most possible ways to effect the disease state, as signaling events mediated by focal adhesion kinase (Fig. 5e).

To validate the microarray data, qRT-PCR was used to analyze the consistency of the results. Six of the 56 differentially expressed lncRNAs were selected: the two upregulated lncRNAs (RP11-629B11.4, HNRNPKP2) and the four downregulated lncRNAs (RP11-770 J1.3, CTD-2260A17.1, RP11-815 J21.2). As shown in Fig. 6b, the trend (upregulation or downregulation) in the change expression of five selected genes were consistent with the microarray analysis results. At the same time, five differentially expressed candidates were validated in DC-SIGNR overexpression cell lines. Identified RP11-629B11.4 and HNRNPKP2 were downregulated with DC-SIGNR overexpression. RP11-770 J1.3, CTD-2260A17.1 and RP11-815 J21.2 were upregulated with DC-SIGNR overexpression. Furthermore to investigated the correlation between DC-SIGNR and HNRNPKP2, the two genes expression were verified by qRT-PCR in 17 paired formalin fixed and paraffin embedded gastric cancer tissues and corresponding para-carcinoma tissues, normalizing to GAPDH. Figure 6c was showed that both DC-SIGNR expression and HNRNPKP2 expression were significantly increased in gastric cancer FFPET compared with para-carcinoma FFPET. Linear regression analysis confirmed that DC-SIGNR expression was negatively correlated with HNRNPKP2 expression in III and IV gastric cancer FFPET (R^2^ = 0.85, P = 0.0004). In the meanwhile, we got the same correlation in I and II gastric cancer FFPET (R^2^ = 0.62, P = 0.02) (Fig. 6d left). However, there was no correlation between DC-SIGNR and HNRNPKP2 in para-carcinoma FFPET both in early stage and middle-late stage (R^2^ = 0.03, P = 0.67; R^2^ = 0.0.0003, P = 0.96). (Fig. 6d right).

### STAT5A promotes HNRNRKP2 expression in gastric cancer cell lines

**Fig. 7:**
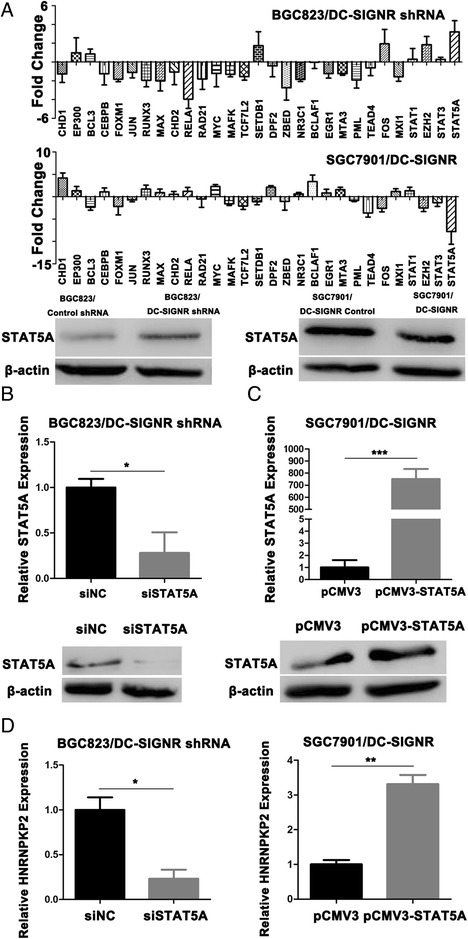
STAT5A promotes HNRNPKP2 expression. **a**, The levels of 29 transcription factors in BGC823 cells after DC-SIGNR knockdown by real-time PCR (up). The levels of 29 transcription factors in SGC7901 cells after DC-SIGNR overexpression by real-time PCR (middle). Western blot confirmed the protein level of STAT5A in BGC823 cells after DC-SIGNR knockdown and in SGC7901 cells after DC-SIGNR overexpression (down). **b**-**c** Real-time PCR confirmed the STAT5A expression and western blot analysis of STAT5A protein level in BGC823 cells after DC-SIGNR knockdown with si-STAT5A and in SGC7901 cells after DC-SIGNR overexpression with pCMV3-STAT5A. D, HNRNPKP2 expression in BGC823 cells after DC-SIGNR with si-STAT5A and in SGC7901 cells after DC-SIGNR overexpression with pCMV3-STAT5A is analyzed by real-time PCR

The enhancers can improve HNRNPKP2 expression and transcription factors binding to enhancers were necessary. The transcription factors binding to enhancers were predicted by searching from GeneCards. Some HNRNPKP2 enhancers binding sites were located upstream or downstream of transcription start site. Within −11.4 to transcription start site and transcription start site to +68.4, we got several transcription factors which had no previous study. Our results showed that STAT5A was significantly increased after knockdown DC-SIGNR in BGC823 cell line and significantly decreased after overexpression DC-SIGNR in SGC7901cell line (Fig. 7a). We next performed knockdown or overexpression STAT5A assays to verify HNRNPKP2 expression. First we conducted transient transfection using si-STAT5A in BGC823 with knockdown DC-SIGNR and STAT5A expression plasmid in SGC7901 with overexpression DC-SIGNR. QRT-PCR results and western blot results verified that mRNA and protein levels of STAT5A were knockdown in BGC823/DC-SIGNR shRNA and overexpression in SGC7901/DC-SIGNR (Fig. 7b and c). Then, qRT-PCR results indicated that HNRNPKP2 expression was decreased with knockdown STAT5A. In contrast, HNRNPKP2 expression was increased with overexpression STAT5A (Fig. 7d). These results suggest that STAT5A is activated after knockdown DC-SIGNR and required to promote HNRNPKP2 expression.

### CXCR4 is responsed to HNRNRKP2

**Fig. 8:**
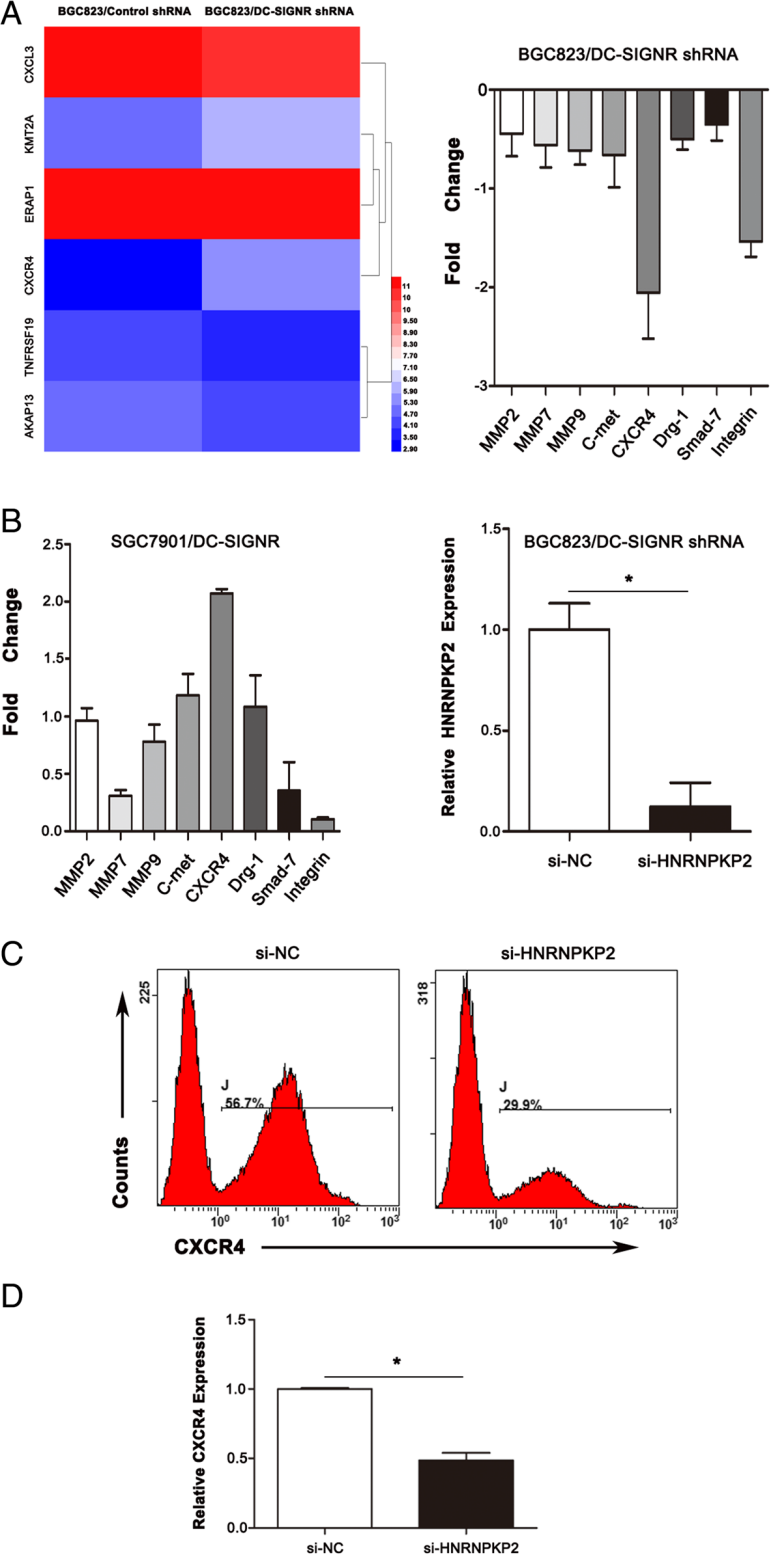
CXCR4 is significantly disregulated after DC-SIGNR knockdown. **a** a heatmap of target genes (CXCL3, KMT2A, ERAP1, CXCR4, TNFRSF19 and AKAP13) after DC-SIGNR knockdown (left panel). The levels of metastasis and invasion markers in BGC823 cells after DC-SIGNR knockdown by qRT-PCR (right panel). **b**,the levels of metastasis and invasion markers in SGC7901 cells after DC-SIGNR overexpression by qRT-PCR. And HNRNPKP2 expression in BGC823 cells after DC-SIGNR and HNRNPKP2 knockdown was analyzed by qRT-PCR. **c** flow cytometry indicated CXCR4 in BGC823 cells after DC-SIGNR and HNRNPKP2 knockdown. **d** CXCR4 expression in BGC823 cells after DC-SIGNR and HNRNPKP2 knockdown was analyzed by qRT-PCR

Microarray data showed that CXCR4 was the target gene of HNRNRKP2 (Fig. 8a). During some genes related with invasion and metastasis, CXCR4 was obviously changed in BGC823 cell lines with DC-SIGNR knockdown and increased in SGC7901 cell lines with DC-SIGNR overexpression (Fig. 8a and b). To investigate the link between HNRNPKP2 and CXCR4 expression, we transfected si-HNRNPKP2 in BGC823 with knockdown DC-SIGNR (Fig. 8b right). QRT-PCR results showed that CXCR4 was decreased after transfection with si-HNRNPKP2 (Fig. 8d). Consistently, flow cytometric analysis exhibited that CXCR4 was significantly downregulated after knockdown HNRNPKP2 48 h (Fig. 8c). Our results revealed that CXCR4 was responded to HNRNRKP2 and represented an important downstream effector of HNRNPKP2 that potentially mediated the effects of this lncRNA on tumour metastasis.

## Discussion

Metastasis accompanies advanced stages of gastric cancer progression. However, the role of selectin in gastric cancer liver metastasis remains poorly understood. We focus on DC-SIGNR which has been associated with tumour biological functions. Therefore, DC-SIGNR has an important role in cancer metastasis. In this research, we explore whether DC-SIGNR mediates gastric cancer liver metastasis.

Our previous research results show that LSECtin of C-type lectin family plays an important role in tumour metastasis [8]. LSECtin has no expression in colon cancer cells but a lot of expression in sinusoidal endothelial cells. During metastasis, some ligands maybe mediate engagement. In this study, we find another C-type lectin family member DC-SIGNR acts as oncogene by promoting cell proliferation, malignant and invasion in gastric cancer metastasis. Unlike colon cancer metastasis, DC-SIGNR expresses in gastric cancer cells mediated liver metastasis. The mechanisms potential the role of DC-SIGNR in metastasis regulation were complex. And which molecule in downstream can respond to DC-SIGNR to contribute to metastasis and invasion in gastric cancer, we focus on long non-coding RNA. Strikingly, Heterogeneous nuclear ribonucleoprotein K pseudogene 2 (HNRNPKP2), as a pseudogene, is found to be upregulated in BGC823 cell line after DC-SIGNR depletion. So far, there has been no related research on HNRNPKP2. Heterogeneous nuclear ribonucleoprotein K (hnRNP K) and HNRNPKP2 are highly homologous. According to reports, pseudogenes act as a antisense regulator to effect on protein-coding mRNAs, such as PTEN [28]. Therefore, we speculate that HNRNPKP2 transcript may have the same function to suppress or enhance hnRNP K. HnRNP K as multifunctional signaling protein has several functions in tumorigenesis. First, hnRNP K interacts with K17 to regulate some pro-inflammatory expression, such as C-X-C chemokine, promoting skin tumour keratinoscyes to grow and invade by CXCR3 signaling pathway [29]. Second, hnRNP K regulates gene expression of proliferation to affect tumour progression, such as c-myc, c-Sre and p53 [30–33]. Third, focal adhesion can integrate internal and external signal to control cells migration and focal adhesion compound precursor contains hnRNP K. The content of hnRNP K plays an important role in metastasis, limiting the speed of cell proliferation [34, 35]. Our results show that most of genes involve in signaling events mediated by focal adhesion kinase. Additionally, the negative correlation between DC-SIGNR and HNRNPKP2 had been found not only in gastric cell lines but also in gastric cancer tissues. From sDC-SIGNR of gastric cancer patients, we speculate DC-SIGNR should be mediated gastric liver metastasis in middle-late stage. Therefore, 17 paired formalin fixed and paraffin embedded gastric cancer tissues and corresponding para-carcinoma tissues can be divided into different stages to analyze if DC-SIGNR effected HNRNPKP2 in middle-late stage. It is better to get more middle-late stage gastric cancer solid tumours to explore the association between DC-SIGNR and HNRNPKP2. Based on them, we speculate that DC-SIGNR mediated gastric cancer liver metastasis maybe causes by HNRNPKP2 to effect on hnRNP K for further studies.

So far, there are some researches on transcription factors to regulate lncRNA expression. STAT3 binds to lncRNA on dendritic cell to effect its differentiation [36]. ANRIL (lncRNA) regulated mTOR and CDK6/E2F1 pathway and then CDK6/E2F1 promotes ANRIL expression in form of positive feedback. At the same time, ANRIL suppresses miR-49a/449a expression in trans by binding to PRC2 which contained EZH2, SUZ12 and EED [37]. Activated by c-Fos at transcription level, MALAT1 (lncRNA) interacts with EZH2 and SUZ12 to increased RCC progression [38]. The numerous CCAT1 (lncRNA) is increased by c-Myc which binding to the E-box on CCAT1 promoter region [39]. So, we speculate that some transcription factors regulated HNRNPKP2 expression with different DC-SIGNR expression. In solid tumors, persistent STAT molecules are involved in tumorigenesis in many cancers by regulating the expression of critical mediators during cancer formation and metastatic progression. That is to say, some gene expression more depends on STAT activity. STAT molecules migrate from cytoplasm to nucleus and occupied upstream specific promoter region to regulate gene expression at transcription level [40, 41]. Similarly, we take effort to find which transcription factor to regulate HRNNPKP2 expression. From some candidate transcription factors, STAT5A can emerge as transcription activator to enhance HNRNPKP2 expression by binding to enhancer. The exact DNA sequences need to be further verified.

Chemokines and their receptors are one of the most representative patterns for organ-selective examples [42]. CXCR4 is a seven-span transmembrane G protein-coupled chemokine receptor and CXC chemokine ligand (CXCL) 12 is its unique ligand. CXCR4 is usually overexpressed in a variety of human cancers [43, 44], but absent or low in a lot of normal tissues [45]. Inflammatory chemokine CXCL12 of enriched organs forms a concentration gradient and attracts CXCR4 positive tumour cells to homing by directed movement. Tumour cells moved towards the highest concentration CXCL12 gradients, realizing the directional migration and positive feedback mechanisms in an autocrine manner [46]. Hence, CXCL12/CXCR4 biological axis plays an important role in regulating specific organs metastasis. After together with its corresponding receptor, there is a transducting signal by intracellular calcium influx and leading to the activation of downstream pathways, such as MAPK1/MAPK3 activation.

## Conclusion

We demonstrate that DC-SIGNR facilitates gastric cancer liver metastasis mediated by HNRNPKP2 regulated by STAT5A via the CXCL12/CXCR4 biological axis.

## Additional files

Additional file 1: **Table S1.** Clinical data of the gastric cancer patients in sDC-SIGNR ELISA study (XLSX 21 kb)

Additional file 2: **Figure S1.** The standard curve of sDC-SIGNR (PNG 24 kb)

Additional file 3: **Table S3.** The names of the genes and sequences of primers (DOCX 22 kb)

Additional file 4: **Table S4.** Target sequences for DC-SIGNR knockdown and overexpression, for HNRNPKP2 siRNA, for STAT5A siRNA and overexpression (DOCX 13 kb)

Additional file 5: **Table S2.** List of lncRNAs regulated by DC-SIGNR (DOC 114 kb)

Additional file 6: **Table S5.** List of target genes regulated by DC-SIGNR (DOCX 12 kb)

## References

[1] Torre LA, Bray F, Siegel RL, Ferlay J, Lortet-Tieulent J, Jemal A (2015). Global cancer statistics, 2012. CA Cancer J Clin.

[2] Ferro A, Peleteiro B, Malvezzi M (2014). Worldwide trends in gastric cancer mortality (1980–2011), with predictions to 2015, and incidence by subtype. Eur J Cancer.

[3] Sakamoto Y, Ohyama S, Yamamoto J (2003). Surgical resection of liver metastases of gastric cancer: an analysis of a 17-year experience with 22 patients. Surgery.

[4] Koga R, Yamamoto J, Ohyama S (2007). Liver resection for metastatic gastric cancer: experience with 42 patients including eight long-term survivors. Jpn J Clin Oncol.

[5] Macdonald JS, Smalley SR, Benedetti J (2001). Chemoradiotherapy after surgery compared with surgery alone for adenocarcinoma of the stomach or gastroesophageal junction. N Engl J Med.

[6] Kakeji Y, Morita M, Maehara Y (2010). Strategies for treating liver metastasis from gastric cancer. Surg Today.

[7] Witz IP (2008). The selectin-selectin ligand axis in tumor progression. Cancer Metastasis Rev.

[8] Zuo Y, Ren S, Wang M (2013). Novel roles of liver sinusoidal endothelial cell lectin in colon carcinoma cell adhesion, migration and in-vivo metastasis to the liver. Gut.

[9] Benekli M, Gullu IH, Tekuzman G (1998). Circulating intercellular adhesion molecule-1 and E-selectin levels in gastric cancer. Br J Cancer.

[10] Khatib AM, Kontogiannea M, Fallavollita L (1999). Rapid induction of cytokine and E-selectin expression in the liver in response to metastatic tumor cells. Cancer Res.

[11] Brodt P, Fallavollita L, Bresalier RS (1997). Liver endothelial E-selectin mediates carcinoma cell adhesion and promotes liver metastasis. Int J Cancer.

[12] Khatib AM, Fallavollita L, Wancewicz EV (2002). Inhibition of hepatic endothelial E-selectin expression by C-raf antisense oligonucleotides blocks colorectal carcinoma liver metastasis. Cancer Res.

[13] Jiang Y, Zhang C, Chen K (2014). The clinical significance of DC-SIGN and DC-SIGNR, which are novel markers expressed in human colon cancer. PLoS One.

[14] Zhao J, Liu Y, Huang G (2015). Long non-coding RNAs in gastric cancer: versatile mechanisms and potential for clinical translation. Am J Cancer Res.

[15] Zhao Y, Guo Q, Chen J (2014). Role of long non-coding RNA HULC in cell proliferation, apoptosis and tumor metastasis of gastric cancer: a clinical and in vitro investigation. Oncol Rep.

[16] Xu TP, Huang MD, Xia R (2014). Decreased expression of the long non-coding RNA FENDRR is associated with poor prognosis in gastric cancer and FENDRR regulates gastric cancer cell metastasis by affecting fibronectin1 expression. J Hematol Oncol.

[17] Hu Y, Wang J, Qian J (2014). Long noncoding RNA GAPLINC regulates CD44-dependent cell invasiveness and associates with poor prognosis of gastric cancer. Cancer Res.

[18] Liu XH, Sun M, Nie FQ (2014). Lnc RNA HOTAIR functions as a competing endogenous RNA to regulate HER2 expression by sponging miR-331-3p in gastric cancer. Mol Cancer.

[19] Li H, Yu B, Li J (2014). Overexpression of lncRNA H19 enhances carcinogenesis and metastasis of gastric cancer. Oncotarget.

[20] Sansone P, Bromberg J (2012). Targeting the interleukin-6/Jak/stat pathway in human malignancies. J. Clin. Oncol..

[21] Zhu BM, Kang K, Yu JH (2012). Genome-wide analyses reveal the extent of opportunistic STAT5 binding that does not yield transcriptional activation of neighboring genes. Nucleic Acids Res.

[22] Sutton A, Friand V, Brule-Donneger S (2007). Stromal cell-derived factor-1/chemokine (C-X-C motif) ligand 12 stimulates human hepatoma cell growth, migration, and invasion. Mol Cancer Res.

[23] Martinez-Nunez RT, Louafi F, Friedmann PS (2009). MicroRNA-155 modulates the pathogen binding ability of dendritic cells (DCs) by down-regulation of DC-specific intercellular adhesion molecule-3 grabbing non-integrin (DC-SIGN). J Biol Chem.

[24] Tserel L, Runnel T, Kisand K (2011). MicroRNA expression profiles of human blood monocyte-derived dendritic cells and macrophages reveal miR-511 as putative positive regulator of Toll-like receptor 4. J Biol Chem.

[25] Faghihi MA, Modarresi F, Khalil AM (2008). Expression of a noncoding RNA is elevated in Alzheimer's disease and drives rapid feed-forward regulation of beta-secretase. Nat Med.

[26] Poliseno L, Salmena L, Zhang J (2010). A coding-independent function of gene and pseudogene mRNAs regulates tumour biology. Nature.

[27] Zhang B, Kirov S, Snoddy J (2005). WebGestalt: an integrated system for exploring gene sets in various biological contexts. Nucleic Acids Res.

[28] Johnsson P, Ackley A, Vidarsdottir L (2013). A pseudogene long-noncoding-RNA network regulates PTEN transcription and translation in human cells. Nat Struct Mol Biol.

[29] Chung BM, Arutyunov A, Ilagan E (2015). Regulation of C-X-C chemokine gene expression by keratin 17 and hnRNP K in skin tumor keratinocytes. J Cell Biol.

[30] Michelotti EF, Michelotti GA, Aronsohn AI (1996). Heterogeneous nuclear ribonucleoprotein K is a transcription factor. Mol Cell Biol.

[31] Ostareck-Lederer A, Ostareck DH, Cans C, et al. c-Src-mediated phosphorylation of hnRNP K drives translational activation of specifically silenced mRNAs. Molecular and cellular biology. 2002;22:4535–4543. 10.1128/MCB.22.13.4535-4543.2002 PMC133888 12052863

[32] Moumen A, Masterson P, O'Connor MJ (2005). hnRNP K: an HDM2 target and transcriptional coactivator of p53 in response to DNA damage. Cell.

[33] Enge M, Bao W, Hedstrom E (2009). MDM2-dependent downregulation of p21 and hnRNP K provides a switch between apoptosis and growth arrest induced by pharmacologically activated p53. Cancer Cell.

[34] de Hoog CL, Foster LJ, Mann M (2004). RNA and RNA binding proteins participate in early stages of cell spreading through spreading initiation centers. Cell.

[35] Inoue A, Sawata SY, Taira K (2007). Loss-of-function screening by randomized intracellular antibodies: identification of hnRNP-K as a potential target for metastasis. Proc Natl Acad Sci U S A.

[36] Wang P, Xue Y, Han Y (2014). The STAT3-binding long noncoding RNA lnc-DC controls human dendritic cell differentiation. Science.

[37] Zhang EB, Kong R, Yin DD (2014). Long noncoding RNA ANRIL indicates a poor prognosis of gastric cancer and promotes tumor growth by epigenetically silencing of miR-99a/miR-449a. Oncotarget.

[38] Hirata H, Hinoda Y, Shahryari V (2015). Long Noncoding RNA MALAT1 Promotes Aggressive Renal Cell Carcinoma through Ezh2 and Interacts with miR-205. Cancer Res.

[39] Yang F, Xue X, Bi J (2013). Long noncoding RNA CCAT1, which could be activated by c-Myc, promotes the progression of gastric carcinoma. J Cancer Res Clin Oncol.

[40] Levine RL, Pardanani A, Tefferi A (2007). Role of JAK2 in the pathogenesis and therapy of myeloproliferative disorders. Nat Rev Cancer.

[41] Silva M, Richard C, Benito A (1998). Expression of Bcl-x in erythroid precursors from patients with polycythemia vera. N Engl J Med.

[42] Mathot L, Stenninger J (2012). Behavior of seeds and soil in the mechanism of metastasis: a deeper understanding. Cancer Sci.

[43] Balkwill F (2004). Cancer and the chemokine network. Nat Rev Cancer.

[44] Balkwill F (2004). The significance of cancer cell expression of the chemokine receptor CXCR4. Semin Cancer Biol.

[45] Sun X, Cheng G, Hao M (2010). CXCL12/CXCR4/CXCR7 chemokine axis and cancer progression. Cancer Metastasis Rev.

[46] Ehling J, Tacke F (2016). Role of chemokine pathways in hepatobiliary cancer. Cancer Lett.

