# Supplementary figures and images for: DC - SIGNR by influencing the lncRNA HNRNPKP2 upregulates the expression of CXCR4 in gastric cancer liver metastasis
Source: Mol Cancer. 2017 Apr 13;16:78. doi: 10.1186/s12943-017-0639-2 (PMC5390362; doi:10.1186/s12943-017-0639-2)

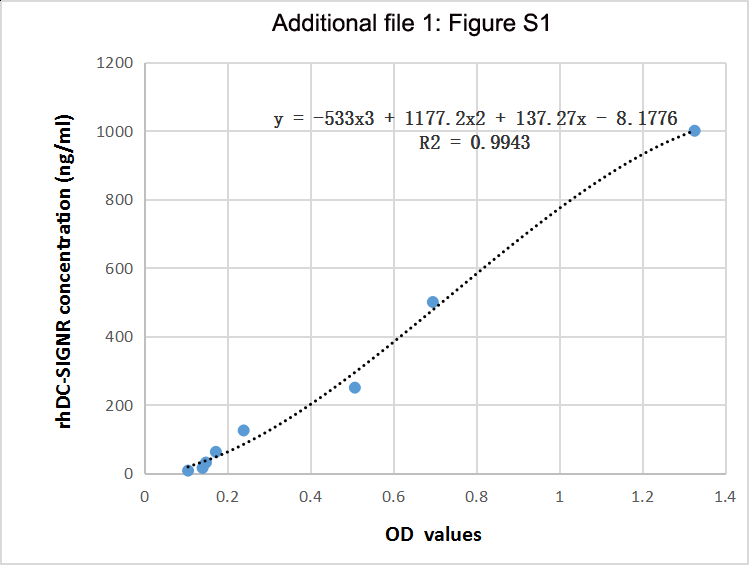

Supplement: Supplementary file 2 — Figure S1. The standard curve of sDC-SIGNR (PNG 24 kb) [file 12943_2017_639_MOESM2_ESM.png]
