# Supplementary material for: DC - SIGNR by influencing the lncRNA HNRNPKP2 upregulates the expression of CXCR4 in gastric cancer liver metastasis
Source: Mol Cancer. 2017 Apr 13;16:78. doi: 10.1186/s12943-017-0639-2 (PMC5390362; doi:10.1186/s12943-017-0639-2)
Supplement: Supplementary file 3 — Table S3. The names of the genes and sequences of primers (DOCX 22 kb) [file 12943_2017_639_MOESM3_ESM.docx]

**Additional file 2: Table S3. The names of the genes and sequences of primers**

| Primary description | Forward (F) and reverse (R) primer sequences |
| --- | --- |
| RP11-815J21.2-F | 5′-TCAAATGCCTGGAATCACCAC-3′ |
| RP11-815J21.2-R | 5′-TGCACGTCACAAGTAAGCCT-3′ |
| RP11-629B11.4-F | 5′-CGTTGGTTCACGACTTGGAAAC-3′ |
| RP11-629B11.4-R | 5′-GCTGACCATGTTTATCCTGATAGAG-3′ |
| HNRNPKP2-F | 5′-GTTTGCTGACGACCGTGGA-3′ |
| HNRNPKP2-R | 5′-GCAACATTCCGAGCTCTGCT-3′ |
| RP11-770J1.3-F | 5′-AGGCGGAGTCGAAAAGACC-3′ |
| RP11-770J1.3-R | 5′-GTCTGGAGCCAAGCACTCTT-3′ |
| CTD-2260A17.1-F | 5′-CGCTTCATTACAGAGGGTTAGTGT-3′ |
| CTD-2260A17.1-R | 5′-ACGCGTATCCTTCTGGCCTA-3′ |
| CXCR4-F | 5′-GCAGCAGGTAGCAAAGTGAC-3′ |
| CXCR4-R | 5′-GCCCATTTCCTCGGTGTAGT-3′ |
| DC-SIGNR-F | 5′-CCAGCAGATACATGGCCACA-3′ |
| DC-SIGNR-R | 5′-CCAGCCAAGAGCATGAAGGA-3′ |
| MMP2-F | 5′-AAGTATGGCTTCTGCCCTGA-3′ |
| MMP2-R | 5′-ATTTGTTGCCCAGGAAAGTG-3′ |
| MMP7-F | 5′-AACTCCCGCGTCATAGAAAT-3′ |
| MMP7-R | 5′-GATACGATCCTGTAGGTGAC-3′ |
| MMP9-F | 5′-TCCTCCCTGGAGACCTGAGA-3′ |
| MMP9-R | 5′-ATTTCGACTCTCCACGCATC-3′ |
| C-met-F | 5′-CAGATGTGTGGTCCTTTG-3′ |
| C-met-R | 5′-ATTCGGGTTGTAGGAGTCT-3′ |
| Drg-1-F | 5′-AGGCGGACATTCTGGAAATG-3′ |
| Drg-1-R | 5′-CGGTACTTCCCCAGCACACTT-3′ |
| Smad-7-F | 5′-TGCTCCCATCCTGTGTGTTAAG-3′ |
| Smad-7-R | 5′-TCAGCCTAGGATGGTACCTTGG-3′ |
| Integrin-F | 5′-GCCTGTGGAGTACAAGTCCTT-3′ |
| Integrin-R | 5′-AATTCGGGTGAAGTTATCTGTGG-3′ |
| CHD1 | 5′-CGATGATAGCAGCAGGGACA-3′ |
| CHD1 | 5′-TGATCCCAATCACGATGGTCT-3′ |
| EP300 | 5′-GCAGGCATGGTTCCAGTTTC-3′ |
| EP300 | 5′-GCCACGGATCATACTTGGGT-3′ |
| BCL3 | 5′-CTGGAGGCCCGCAATTATGA-3′ |
| BCL3 | 5′-AATGTCCACTGCGTCGATGT-3′ |
| CEBPB | 5′-GCCGGTTTCGAAGTTGATGC-3′ |
| CEBPB | 5′-GACAGTTACACGTGGGTTGC-3′ |
| FOXM1 | 5′-CATAGCAAGCGAGTCCGCAT-3′ |
| FOXM1 | 5′-TAGCAGCACTGATAAACAAAGAAAG-3′ |
| RAD21 | 5′-CAGCCACTGCCTGACTTAGA-3′ |
| RAD21 | 5′-GCTGGTGCTCTGTTCAGACT-3′ |
| RUNX3 | 5′-TTCAACGACCTTCGCTTCGT-3′ |
| RUNX3 | 5′-CCTTGATGGCTCGGTGGTAG-3′ |
| MAX | 5′-GAGGTTTCAATCTGCGGCTG-3′ |
| MAX | 5′-TCTTGCTGGTGTGTGTGGTT-3′ |
| CHD2 | 5′-AGTCGTCCCTTTATCCACCCT-3′ |
| CHD2 | 5′-CTGCCCAGCACAGTCTTATCT-3′ |
| RELA | 5′-CGCATCCAGACCAACAACAA-3′ |
| RELA | 5′-AGATCTTGAGCTCGGCAGTG-3′ |
| STAT3 | 5′-GGAGCATCCTGAAGCTGACC-3′ |
| STAT3 | 5′-GTAGGCGCCTCAGTCGTATC-3′ |
| JUN | 5′-GGAGACAAGTGGCAGAGTCC-3′ |
| JUN | 5′-CCAAGTTCAACAACCGGTGC-3′ |
| MYC | 5′-CAGCGACTCTGAGGAGGAAC-3′ |
| MYC | 5′-CCCTCTTGGCAGCAGGATAG-3′ |
| MAFK | 5′-GGATGCAGCTGGCTTGTAGA-3′ |
| MAFK | 5′-CACGAAGCTCTCCTGGACTG-3′ |
| TCF7L2 | 5′-CGGCCATCAACCAGATCCTT-3′ |
| TCF7L2 | 5′-GCCCGACACTTCTTTGGAGT-3′ |
| SETDB1 | 5′-AACATCCTCAGCCTCTGCAC-3′ |
| SETDB1 | 5′-AGTCACTGTCACCTGCTTGG-3′ |
| DPF2 | 5′-AGGCAGAGGAACAGGGAAGA-3′ |
| DPF2 | 5′-CTCCGGTCTGTGAGTCCAAG-3′ |
| ZBED1 | 5′-TTCCTGGAGCCAGCAGGT-3′ |
| ZBED1 | 5′-TCTCCAGGTGGTAGGACAGG-3′ |
| EZH2 | 5′-AAGGGCACAGCAGAAGAACT-3′ |
| EZH2 | 5′-TTGCACTTACGATGTAGGAACC-3′ |
| NR3C1 | 5′-GCAGTGGAAGGACAGCACAA-3′ |
| NR3C1 | 5′-CCTCCAACAGTGACACCAGG-3′ |
| BCLAF1 | 5′-AGAAGCGATACAGTTCTAGGTC-3′ |
| BCLAF1 | 5′-ACTCCTGGAACGTGAACGAC-3′ |
| EGR1 | 5′-CCAACAGTGGCAACACCTTG-3′ |
| EGR1 | 5′-GTGGGTTGGTCATGCTCACT-3′ |
| MTA3 | 5′-GTTGAGGCTGACTTGACCGA-3′ |
| MTA3 | 5′-TGTTGCGGGCAGAGATTCAT-3′ |
| PML | 5′-AGCAGTGAGTCCAGTGACCT-3′ |
| PML | 5′-GGACACGGCCTTGGAGTAGA-3′ |
| STAT5A | 5′-ACCTGTGGTTGTCATCGTCC-3′ |
| STAT5A | 5′-TTTGTCAGGCACGGCAAATG-3′ |
| TEAD4 | 5′-ATGATGAACAGCGTGCTGGA-3′ |
| TEAD4 | 5′-CAGCAAGGTCTCCTGTGTGT-3′ |
| FOS | 5′-CCTAACCGCCACGATGATGT-3′ |
| FOS | 5′-TGAAGTTGGCACTGGAGACG-3′ |
| MXI1 | 5′-CCGCCCGTCGCACAT-3′ |
| MXI1 | 5′-GACGGGAATGAAGAGGCGTAG-3′ |
| STAT1 | 5′-TGGATCAGCTGCAGAACTGG-3′ |
| STAT1 | 5′-GAAGGTGCGGTCCCATAACA-3′ |
| GAPDH-F | 5′-CCTCAAGATCATCAGCAAT-3′ |
| GAPDH-R | 5′-CCATCCACAGTCTTCTGGGT-3′ |
