# Supplementary material for: DC - SIGNR by influencing the lncRNA HNRNPKP2 upregulates the expression of CXCR4 in gastric cancer liver metastasis
Source: Mol Cancer. 2017 Apr 13;16:78. doi: 10.1186/s12943-017-0639-2 (PMC5390362; doi:10.1186/s12943-017-0639-2)
Supplement: Supplementary file 4 — Table S4. Target sequences for DC-SIGNR knockdown and overexpression, for HNRNPKP2 siRNA, for STAT5A siRNA and overexpression (DOCX 13 kb) [file 12943_2017_639_MOESM4_ESM.docx]

**Additional file 2: Table S4. target sequences for DC-SIGNR knockdown and overexpression, for HNRNPKP2 siRNA, for STAT5A siRNA and overexpression**

| Name | Sequences |
| --- | --- |
| sh-DC-SIGNR1 | 5′-GCTCCCTAAGTCAGGAACAAT-3′ |
| sh-DC-SIGNR2 | 5′-GGACTGGACATTCTTCCAAGG-3′ |
| sh-NC | 5′-TTCTCCGAACGTGTCACGTTTC-3′ |
| overexpression DC-SIGNR, transcript variant 8 | http://www.ncbi.nlm.nih.gov/nuccore/NM_001144910.1 |
| overexpression STAT5A | NM_003152.2 |
| si-HNRNPKP2 sense | 5′-CCAGGAAUGUUGUCCUCAUTT-3′ |
| si-HNRNPKP2 antisense | 5′-AUGAGGACAACAUUCCUGGTT-3′ |
| si-STAT5A sense | 5′-CCGGCACAUUCUGUACAAUTT-3′ |
| si-STAT5A antisense | 5′-AUUGUACAGAAUGUGCCGGTT-3′ |
| si-NC sense | 5′-UUCUCCGAACGUGUCACGUTT-3′ |
| si-NC antisense | 5′-ACGUGACACGUUCGGAGAATT-3′ |
