# Supplementary material for: DC - SIGNR by influencing the lncRNA HNRNPKP2 upregulates the expression of CXCR4 in gastric cancer liver metastasis
Source: Mol Cancer. 2017 Apr 13;16:78. doi: 10.1186/s12943-017-0639-2 (PMC5390362; doi:10.1186/s12943-017-0639-2)
Supplement: Supplementary file 5 — Table S2. List of lncRNAs regulated by DC-SIGNR (DOC 114 kb) [file 12943_2017_639_MOESM5_ESM.doc]

**Additional file 2: Table S2. List of lncRNAs regulated by DC-SIGNR**

| **ProbeName** | **p-value** | **Fold change** | **regulation** | **seqname** | **GeneSymbol** |
| --- | --- | --- | --- | --- | --- |
| ASPWP0151594 | 0.030556436 | 4.7621621 | up | ENST00000514414 | RP11-629B11.4 |
| ASPWP0007032 | 0.019362334 | 4.3702522 | down | ENST00000556583 | RP11-770J1.3 |
| ASPWP0172079 | 0.087787589 | 4.1917963 | up | ENST00000564977 | RP11-395B7.7 |
| ASPWP0005421 | 0.032775276 | 4.0580366 | down | ENST00000512856 | CTD-2260A17.1 |
| ASPWP0154293 | 0.01569933 | 4.0472515 | down | ENST00000514756 | CCNG2 |
| ASPWP0002881 | 0.006237421 | 3.9928944 | down | ENST00000561409 | RP11-815J21.2 |
| ASPWP0183295 | 0.000697636 | 3.8075603 | up | ENST00000419045 | AC115617.2 |
| ASPWP0131110 | 0.009508846 | 3.7711125 | up | ENST00000447355 | HNRNPKP2 |
| ASPWP0006423 | 0.384060812 | 3.7194017 | down | ENST00000456356 | RP11-229P13.23 |
| ASPWP0005600 | 0.312232736 | 3.5313804 | down | ENST00000455534 | MYB-AS1 |
| ASPWP0006743 | 0.034117398 | 3.3067608 | down | ENST00000427492 | RP11-537A6.9 |
| ASPWP0097179 | 0.104712506 | 3.1488103 | up | ENST00000552169 | SETP7 |
| ASPWP0006844 | 0.210301841 | 3.1472811 | up | ENST00000529475 | RP11-438N5.3 |
| ASPWP0100015 | 0.276071729 | 3.0178834 | down | TCONS_00007535 | XLOC_003546 |
| ASPWP0007410 | 0.00163322 | 2.9946917 | down | uc003myx.3 | LOC100130275 |
| ASPWP0183849 | 0.034867866 | 2.876356 | up | ENST00000412087 | HNRNPA1P27 |
| ASPWP0007377 | 0.00708178 | 2.8754724 | down | NR_015450 | LOC285548 |
| ASPWP0196130 | 0.022952932 | 2.831234 | up | ENST00000325243 | CBX3P1 |
| ASPWP0129528 | 0.017289296 | 2.7929471 | up | ENST00000418415 | AC016912.3 |
| ASPWP0007265 | 0.013092756 | 2.6615864 | down | ENST00000445745 | AC016683.6 |
| ASPWP0004531 | 0.05949585 | 2.6353405 | down | ENST00000422408 | RP3-508I15.9 |
| ASPWP0006932 | 0.055274823 | 2.6336188 | up | ENST00000534148 | H2AFZP4 |
| ASPWP0002557 | 0.00376246 | 2.6009801 | up | ENST00000418340 | RPL35AP31 |
| ASPWP0113233 | 0.039630532 | 2.5936592 | down | ENST00000565374 | RP11-2L4.1 |
| ASPWP0106782 | 0.019777305 | 2.5904722 | up | ENST00000503999 | AC084854.4 |
| ASPWP0003181 | 0.028164137 | 2.5809324 | down | NR_003574 | ABCA17P |
| ASPWP0007438 | 0.137648518 | 2.5102185 | down | ENST00000520891 | RP3-399L15.3 |
| ASPWP0002349 | 0.236873623 | 2.4882537 | down | TCONS_00007321 | XLOC_003354 |
| ASPWP0144615 | 0.047133587 | 2.4609344 | up | ENST00000444020 | RP11-1029M24.1 |
| ASPWP0004449 | 0.229395811 | 2.4575654 | down | uc002yoz.1 | BC041449 |
| ASPWP0005405 | 0.108782194 | 2.4503761 | down | uc001ezm.1 | AC2 |
| ASPWP0007266 | 0.017454685 | 2.4239264 | up | NR_047570 | LOC654433 |
| ASPWP0007409 | 0.001326399 | 2.4153669 | up | ENST00000505151 | CTC-338M12.4 |
| ASPWP0002648 | 0.133410136 | 2.4058639 | down | ENST00000560419 | RP11-293M10.6 |
| ASPWP0002768 | 0.260746624 | 2.3022201 | up | uc001ygz.2 | BC014138 |
| ASPWP0178019 | 0.054518706 | 2.2966148 | down | ENST00000517606 | RP11-128L5.1 |
| ASPWP0002154 | 0.008317784 | 2.2910661 | up | ENST00000433407 | RP11-312B8.2 |
| ASPWP0006843 | 0.016981727 | 2.2876214 | up | ENST00000308739 | AC090804.1 |
| ASPWP0164759 | 0.004809771 | 2.2795905 | down | ENST00000439406 | HCG20 |
| ASPWP0190341 | 0.06475868 | 2.1941891 | up | ENST00000391458 | HMGN2P8 |
| ASPWP0114045 | 0.348328353 | 2.1875315 | down | TCONS_00017515 | XLOC_008172 |
| ASPWP0191099 | 0.014910646 | 2.1688106 | up | ENST00000231383 | RP11-179H18.4 |
| ASPWP0006344 | 0.028402402 | 2.166063 | up | ENST00000366466 | AL353671.3 |
| ASPWP0092546 | 0.230179195 | 2.1499278 | down | ENST00000536217 | RP11-366L20.4 |
| ASPWP0000829 | 0.010847273 | 2.1431728 | down | ENST00000463353 | RP11-331F4.4 |
| ASPWP0119031 | 0.182353486 | 2.1376276 | down | TCONS_00018642 | XLOC_008993 |
| ASPWP0103441 | 0.006637695 | 2.1258671 | up | ENST00000554535 | RP11-561B11.4 |
| ASPWP0100941 | 0.060151952 | 2.1253784 | up | ENST00000547436 | BTF3P2 |
| ASPWP0144512 | 0.05452315 | 2.1202081 | up | ENST00000431490 | RP11-578F5.1 |
| ASPWP0090580 | 0.183407415 | 2.1106362 | down | TCONS_00004454 | XLOC_002331 |
| ASPWP0108995 | 0.169971271 | 2.0753541 | up | ENST00000486386 | CTD-2184D3.1 |
| ASPWP0176024 | 0.157412118 | 2.0638358 | up | ENST00000411586 | RP11-118H4.1 |
| ASPWP0007820 | 0.017640309 | 2.048487 | down | ENST00000467381 | RP11-331F4.4 |
| ASPWP0187669 | 0.011274173 | 2.0348282 | up | ENST00000422205 | TMEM14D |
| ASPWP0003939 | 0.007933534 | 2.0309701 | up | ENST00000439412 | AC010733.4 |
| ASPWP0003900 | 0.308474251 | 2.0221488 | down | ENST00000439180 | MYCNOS |
