# Supplementary material for: DC - SIGNR by influencing the lncRNA HNRNPKP2 upregulates the expression of CXCR4 in gastric cancer liver metastasis
Source: Mol Cancer. 2017 Apr 13;16:78. doi: 10.1186/s12943-017-0639-2 (PMC5390362; doi:10.1186/s12943-017-0639-2)
Supplement: Supplementary file 6 — Table S5. List of target genes regulated by DC-SIGNR (DOCX 12 kb) [file 12943_2017_639_MOESM6_ESM.docx]

**Additional file 2: Table S5. List of target genes regulated by DC-SIGNR**

| **ProbeName** | **p-value** | **Fold change** | **regulation** | **seqname** | **GeneSymbol** |
| --- | --- | --- | --- | --- | --- |
| ASPWP0005054 | 0.021529506 | 1.9828602 | down | NM_002090 | CXCL3 |
| ASPWP0001987 | 0.197247696 | 1.812261 | up | NM_001197104 | KMT2A |
| ASPWP0000493 | 0.259165808 | 1.4014586 | up | NM_001040458 | ERAP1 |
| ASPWP0012459 | 0.144608468 | 1.8124345 | down | NM_018647 | TNFRSF19 |
| ASPWP0012621 | 0.031946608 | 2.0756935 | down | NM_007200 | AKAP13 |
| ASPWP0004010 | 0.004180815 | 6.9510278 | down | NM_003467 | CXCR4 |
